# Head-Mounted Display-Based Therapies for Adults Post-Stroke: A Systematic Review and Meta-Analysis

**DOI:** 10.3390/s21041111

**Published:** 2021-02-05

**Authors:** Guillermo Palacios-Navarro, Neville Hogan

**Affiliations:** 1 Department of Electronic Engineering and Communications, University of Zaragoza, 44003 Teruel, Spain; 2 Department of Mechanical Engineering, Massachusetts Institute of Technology, Cambridge, MA 02139, USA; neville@mit.edu; 3 Department of Brain and Cognitive Sciences, Massachusetts Institute of Technology, Cambridge, MA 02139, USA

**Keywords:** head-mounted display, immersive virtual reality, motor recovery, rehabilitation, stroke

## Abstract

Immersive virtual reality techniques have been applied to the rehabilitation of patients after stroke, but evidence of its clinical effectiveness is scarce. The present review aims to find studies that evaluate the effects of immersive virtual reality (VR) therapies intended for motor function rehabilitation compared to conventional rehabilitation in people after stroke and make recommendations for future studies. Data from different databases were searched from inception until October 2020. Studies that investigated the effects of immersive VR interventions on post-stroke adult subjects via a head-mounted display (HMD) were included. These studies included a control group that received conventional therapy or another non-immersive VR intervention. The studies reported statistical data for the groups involved in at least the posttest as well as relevant outcomes measuring functional or motor recovery of either lower or upper limbs. Most of the studies found significant improvements in some outcomes after the intervention in favor of the virtual rehabilitation group. Although evidence is limited, immersive VR therapies constitute an interesting tool to improve motor learning when used in conjunction with traditional rehabilitation therapies, providing a non-pharmacological therapeutic pathway for people after stroke.

## 1. Introduction

In the past decades, there has been an sharp increase in the application of virtual reality (VR) treatments to the rehabilitation of a range of disorders resulting from lesions of the nervous system [1,2]. The area of rehabilitation of patients with stroke is the most productive in terms of technology-based interventions in both upper and lower extremities [3]. VR therapies have been successfully used after stroke [4,5] since they apply concepts that are relevant to stroke rehabilitation, such as high repetition, high intensity, and task-oriented training [6,7].

Virtual reality can provide an engaging and motivational experience, allowing the user to practice motor movements while manipulating an interface device [5]. The virtual environment (VE) can be easily changeable, allowing the design of individualized therapies that are adapted to patient needs. VR can provide functional, rich stimuli (cues) and motivating context (feedback), encouraging the more active participation of the subject [8]. Furthermore, different studies have reported improvements in motor abilities, as well as a great level of participant motivation after including virtual reality in stroke rehabilitation [9,10,11,12,13,14,15]. In stroke rehabilitation, providing an intervention that is motivating and engaging is crucial to patient involvement and participation. Semi-immersive and non-immersive VR systems have been widely used for stroke patient rehabilitation.

### Immersive vs. Non-Immersive VR

Weiss et al. defined virtual reality as “the use of interactive simulations to provide the users with opportunities to engage in environments that appear and feel similar to real-world objects and events” [16]. Schultheis and Rizzo considered virtual reality to be ‘‘an advanced form of human–computer interface that allows the user to interact with and become immersed in a computer-generated environment in a naturalistic fashion” [17]. Regardless of its definition, the multisensorial experience offers a practice environment that can be ecologically valid and has the potential to enhance patient enjoyment and compliance [18], important factors in successful rehabilitation [5,19]. The variety of technologies that are considered VR is broad. We consider technologies ranging from non-immersive applications to completely immersive applications, progressing through semi-immersive ones. It all depends on the degree of isolation from the physical world that the technology provides to the user when interacting with the virtual environment [7].

Lange et al. stated that “immersive VR can be produced by combining computers, head-mounted displays (HMDs), body tracking sensors, specialized interface devices and real-time graphics to immerse a participant in a computer-generated simulated world that changes in a natural way with head and body motion” [20]. A fully immersive VR system is an HMD in which the subject sees only the computer-generated image, and the rest of the physical world is blocked from view [21]. Immersive VR systems often take advantage of visual, auditory, or haptic devices [22,23]. Crosbie et al. also considered immersive VR applications that typically use HMDs [24].

In addition to the immersive mode, VR also comes in a “non-immersive” or “semi-immersive” form. Both modes involve the user’s perception of both the real world and the virtual one simultaneously, but the user is not completely immersed in the virtual environment [25,26]. According to Lange et al., non-immersive modes are commonly generated by using computers and console game systems (as well as nongame lab-generated systems), and the developed therapies are described as game-based rehabilitation, therapy gaming, digital game-based therapy, etc. [20]. Here, the visual aspects of the computer-generated VR environment are presented on a conventional computer monitor or projected onto screens. The subject controls his/her movement within the environment by means of a joystick or other control device.

Immersion and presence are two important aspects of a subject’s performance of virtual tasks [7,24,27,28]. Thus, whereas immersion is a “technology-related” [16], objective aspect of VEs, presence is a psychological, perceptual, and cognitive consequence of immersion. Presence is thought of as the psychological perception of “being in” or “existing in” the VE in which one is immersed [29,30]. The use of HMDs may affect the sense of presence and immersion [31] and, as a result, impact participant motivation to exercise.

In spite of the fact that most of the recently conducted meta-analyses have shown improvements when comparing non-immersive VR systems to conventional therapies [32,33,34,35], there are still analyses that showed a remarkable divergence in the outcome measures used in the included studies [36]. Although some limitations of conventional rehabilitation may have been overcome by the advantages of VR training, limited evidence is available on the clinical effectiveness of immersive VR systems for stroke rehabilitation.

For the purposes of this review, we take into account VR systems for stroke that allow the user to be fully immersed in the virtual environment (VE) by means of an HMD. The following meta-analysis sought to answer the following question: Do HMD-based virtual reality therapies improve motor function more than the same duration of traditional rehabilitation in people after stroke?

## 2. Methods

### 2.1. Eligibility Criteria

We included studies that examine the effects of immersive VR on motor rehabilitation for post-stroke adults. In order to be included in our meta-analysis, the studies had to fulfill the following selection criteria: (a) the study had to apply a VR intervention to a sample of post-stroke adult subjects via a fully immersive device (HMD); (b) the study had to include a control group (conventional therapy or another non-immersive VR intervention, even interventions applied to healthy subjects whenever they served as controls); therefore, pretest–posttest one-group designs and *N* = 1 designs were excluded; (c) the study had to include significant outcomes measuring functional or motor recovery of either lower or upper limbs; (d) the study had to report statistical data for the groups involved in at least the posttest (means, standard deviations, *t* tests, ANOVAs, etc.); (e) the sample size of each group should not be less than 5 subjects in the posttest; (f) the study had to be published in a peer-reviewed journal and written in English. Only randomized controlled trial (RCT) studies were included in an attempt to raise the standard methodological quality. We also excluded studies intended for post-stroke cognitive rehabilitation despite their use of an HMD, as well as any kind of virtual-reality-based contribution developed for recreational or educational purposes.

### 2.2. Search Procedure

We searched the following scientific databases through their online search engines: MEDLINE (Medical Literature Analysis and Retrieval System Online, Bethesda, MD, USA) through PubMed, Cochrane CENTRAL (Central Register of Controlled Trials, Hoboken, NJ, USA), Cochrane Database of Systematic Reviews, and Physiotherapy Evidence Database (PEDro, Newtown, New South Wales, Australia). Studies were collected from inception up to 31 October 2020. In an attempt to identify further relevant studies, reference lists from the identified publications were also reviewed to identify additional research articles of interest or relevant articles that may have been missed during the initial database searches. We initially developed search strategies for Medline and then we adapted them for use in other databases. The following outlines the complete combination of search terms that was used to search the titles and abstracts of potential papers for MEDLINE (Bethesda, MD, USA) and adapted to search the other databases: ((virtual rehabilitation OR virtual reality OR augmented reality OR computer gam* OR virtual gam* OR serious gam* OR video gam* OR augmented gam*) AND (Stroke)) NOT cognitive.

### 2.3. Methodological Quality Assessment

The methodological quality of the studies was rated with the PEDro scale [37,38], which assesses 11 criteria. Specifically, a study is given a point for each of the following: random assignment of subjects; concealed allocation; measures of subject comparability provided at baseline; blinding of subjects; blinding of clinicians administering the interventions; blinding of the assessors; outcome measures obtained from at least 85% of subjects; all subjects for whom outcome measures were available received treatment or control conditions as allocated, or data for at least one outcome were analyzed by “intention to treat” analysis; results of between-group statistical comparisons report on at least one key outcome; and point measures and measures of variability in the outcomes were provided for at least one key outcome. The eligibility criterion item does not contribute to the total score, so the PEDro total score varies from 0 to 10, and the higher the score, the better the methodological quality of the clinical trial.

### 2.4. Quantitative Analysis

To compute the effect size across the studies, we used the standardized mean difference (d), defined as the difference between the means of the experimental group and the control group, both taken in the posttest, divided by a pooled estimate of the within-study standard deviation and corrected by a factor for small samples, as stated by Hedges and Olkin [39]. The magnitude of an effect size is an estimate of the degree to which intervention and control groups differ in terms of their therapeutic effectiveness [40]. Positive values of d indicate a favorable outcome to the intervention. According to Cohen [41], an effect size of d = 0.20 is seen as small, d = 0.50 is medium, and d = 0.80 is large. In the same way, an effect size of d = 2.00 is considered very large according to Sawilowsky [42].

In each study, a different d index was calculated for the outcome measures of the timed and up and go test (TUG), Berg balance scale (BBS), and other measures. Therefore, in the same study, we could calculate more than one d index. The results from comparable trials were pooled by means of the Revman software v5.3 (RevMan; Cochrane, London, UK). Outcomes were directly obtained from each article or converted to meters per second (m/s) from the reported results. Each d index was treated separately according to the outcome measure (by performing separated meta-analyses). In each meta-analysis, a random-effects model was applied, according to which the d index in the posttest was weighted by its inverse variance. The process of analysis consisted of calculating the mean effect size with its 95% confidence interval, assessing heterogeneity (by means of the heterogeneity test Q), and determining the I^2^ index to evaluate the degree of homogeneity of the effect sizes around the average effect [43]. We take into account the classification of I^2^ proposed by Higgins and Thompson [43] by stating that I^2^ values of around 25%, 50%, and 75% can be considered as reflecting small, medium, and large heterogeneity, respectively [44]. When the heterogeneity Q test is statistically significant (*p* < 0.05) and the I^2^ index is at least 50%, then we can assume that the individual effect sizes are heterogeneous. The influence of moderating variables should be taken into account to explain this fact. Since we only included published studies, we may have had important effect sizes (ESs) due to publication bias. This problem occurs when studies reporting large ESs are published and studies reporting null results or smalls ESs are not. Publication bias was investigated by inspecting funnel plots and regression asymmetry tests.

### 2.5. Outcome Measures

The main outcomes found in the studies were the following. For the lower limb: timed up and go (TUG) test, Berg balance scale (BBS), six-minute walk test (6MWT), 10-m walk test (10MWT), functional reach test (FRT), and activities-specific balance confidence (ABC). On the other hand, the outcome measures for the upper limb were the action research arm test (ARAT), the upper extremity motricity index, and the Fugl-Meyer upper extremity test (FMUE). Furthermore, parameters such as cadence, stride length, velocity, and step length were included in some studies.

## 3. Results

### 3.1. Search Results

**Figure 1:**
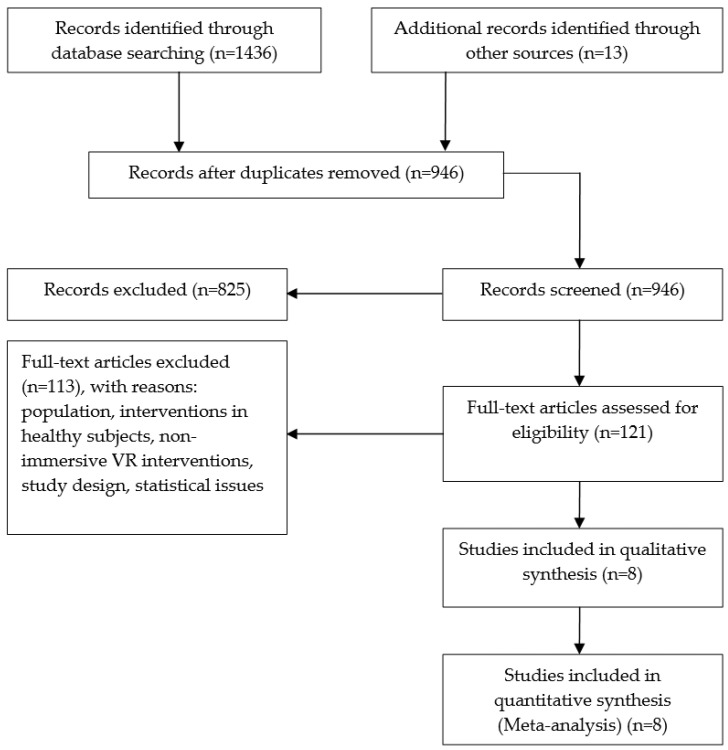
Consort diagram of study selection.

The initial search yielded 1436 articles. We also identified additional records from other sources (13). After removing duplicates, 946 articles that potentially investigated immersive VR interventions were identified. The authors independently evaluated titles and abstracts taking into account the inclusion and exclusion criteria. Due to the fact that, on many occasions, words such as “immersive” or “HMD” were located in neither titles nor abstracts, we had to carry out a deeper screening of potential articles (121). Finally, the population of our study consisted of eight articles. All of them refer to the impact of immersive VR therapies in stroke populations. The details of the search result are summarized in Figure 1.

### 3.2. Methodological Quality Assessment

The PEDro scores for each criterion are presented together with the characteristics of the studies (Table 1). The rankings of all studies but one (Ögün [45]) are available in the PEDro database [37]. The score of the study of Ögün [45] is not yet in PEDro, but the score was obtained by consensus between the authors. The eligibility criteria were clearly satisfied in all of the studies, with the exception of the study of Lee [46], whereas baseline comparability was satisfied in all of the studies. It has to be taken into account that, due to the nature of this type of study, therapists and/or technicians who administer and supervise the interventions know which subjects belong to both control and experimental groups. This means that it is not possible to apply some of the items in the PEDro scale (blinding of subjects and clinicians). In fact, none of the studies fulfilled these two requirements simultaneously. The study of Ögün [45] had patients masked by using sham VR therapy within the control group. The same applies to item 3 (concealed allocation), which was fulfilled by only two studies [47,48]. For all of the above, studies with PEDro scores of 4 or higher were considered to be of a reasonable quality in this review. In addition, our average PEDro score of 5.75 suggests that the included studies were of moderate methodological quality [49]. It is important to note that one study [47] reached the maximum, and two studies reached 7 out of 8 [46,48]. Five studies reported that the outcome assessors were blinded [45,46,47,48,50]. Five studies reported withdrawals and provided reasons for these dropouts [45,46,47,48,51].

### 3.3. Overview of Interventions

After selecting the articles, we extracted the following variables: year of publication, sample size (age and gender), stroke onset, study design, interventions, outcome measures, and conclusions. The details of the general characteristics of the different studies are summarized in Table 1.

The included studies took place in four countries: five trials took place in Korea [46,48,50,51,53], one in the USA [52], one in UK [47], and one in Turkey [45]. Two hundred and thirty-eight participants were included in these trials, with a dropout of 35 subjects. The mean age of participants ranged from 46 to 66 years, and about 60% of them were male. Sample sizes were small in general, ranging from 8 to 32 participants per group. There was also considerable variability in the average month post-stroke (average value ranging between 9 and 140 months).

The duration of interventions varied across studies: from two weeks [52] to eight weeks [51]. One study was conducted in six weeks [45]. Three of the studies were conducted in four weeks [46,48,53], whereas Jung’s [50] and Crosbie’s [47] interventions lasted three weeks. As far as the experiment settings are concerned, all of the studies with the exception of Kang’s study [48] (clinical environment) were carried out in research laboratories. The frequency of interventions varied from 3 to 5 days a week, and the duration of sessions ranged from 20 to 90 min. The total time scheduled for therapy ranged from 1680 min [51] to 270 min [47].

Regarding the completeness of the interventions, Kim et al. [51] reported 10 dropouts (out of 38 subjects) due to the fact that some of them were discharged from the hospital and others were not active enough. Ögün et al. [45] reported 19 dropouts (out of 84) due to compliance issues. One participant dropped out of one treatment session in the study of Crosbie et al. [47]. In the study of Kang et al. [48], two subjects withdrew because of a lack of participation. Finally, Lee et al. [46] reported 3 dropouts out of 21, but an intention-to-treat analysis was performed.

Two studies were intended for upper limb rehabilitation [45,47], whereas six studies were intended for lower limb rehabilitation [46,48,50,51,52,53]. As a rule, studies compared the performance of an experimental group (receiving the VR treatment) to that of a control group (receiving conventional therapies). However, Kim et al. in their study added extra training in the experimental groups compared to the control group [51]. Lee et al. [46] also added extra training for subjects in the experimental group by means of the VR intervention. With the exception of two studies [48,51], the interventions were carried out on two groups. In the study of Kang et al. [48], the immersive VR group was provided with speed-modulated optic flow together with treadmill training. Its performance was compared to a group receiving treadmill training and another group receiving conventional therapy. In the study of Kim [51], three groups performed treadmill gait training, but functional electrical stimulation (FES) was received by the immersive VR group and one of the treadmill groups. Half of the studies used treadmill systems for rehabilitation [48,50,51,52]. The VR interventions of Park et al. [53] and Lee et al. [46] were intended for postural training.

Table 1 presents the general characteristics of the different studies: year of publication, participant age and gender, sample size, stroke onset, study design, intervention procedures, outcome measures, and main findings.

### 3.4. Outcomes Measures and Effect Sizes

A majority of studies used the timed up and go test (TUG) to quantify balance and gait progress [46,48,50,51]. Five studies measured locomotion function: two used the 10MWT [48,53], two used the 6-min walk test (6MWT) [48,52], and two measured spatiotemporal gait ability parameters [46,52,53]. Two studies measured balance via the Berg balance scale (BBS) [46,51]. Walking speed was obtained from reported measures in three studies [46,52,53]. The ABC scale was measured in the study of Jung et al. [50], whereas the functional reach test (FRT) was measured in the study conducted by Kang [48]. The ARAT and upper limb motricity index tests were measured in the study of Crosbie [47]. The primary outcome measure in the study of Ögün et al. [45] was assessed with the Fugl-Meyer upper extremity (FMUE), whereas the ARAT and the functional independence measure (FIM) were used as secondary outcomes. Gait variables such as walking velocity, cadence, and stride length were measured using several electrical spatial and temporal analysis systems [46,53]. As far as follow-up periods are concerned, three studies reported data after two weeks [52], one month [53], and six weeks [47]. Below, we briefly describe the studies included in the analysis.

The study of Jaffe et al. [52] evaluated two interventions aimed at improving certain gait parameters. The task required stepping over objects (for both groups). The control group subjects had to step over foam obstacles along a hallway (they were secured by a gait-belt), whereas subjects in the VR group walked on a treadmill at a self-selected walking speed (they were secured by a harness). Real obstacles were used for the control group, whereas virtual stationary images of obstacles were used in the experimental group (through an HMD). In addition, virtual stationary images of obstacles were introduced. According to the subjects’ opinions, the visual cue obtained thanks to the lateral view of the legs provided an extra advantage in the performance, namely, the position of their feet, the monitoring of the knee flexion, and the control of their stepping height and length, among others. Although both interventions were effective in improving gait parameters (walking endurance, obstacle clearance capacity, gait velocity, and stride length), the VR training produced greater improvements, especially during fast-speed walking.

The study of Kang et al. [48] tried to examine the effects of a modulation in the optic flow speed to provide an effective training method for balance and gait rehabilitation in stroke patients. The design included three groups: the treadmill with optic flow group, the treadmill group, and the control group. They demonstrated that treadmill training with optic flow speed modulation improved the balance and gait in a significant way for the population with chronic stroke. The treadmill with optic flow group showed significant improvement in the timed up and go test, 10-m walk test, and six-minute walk test compared to the treadmill and control groups post-treatment.

Park et al. tried to determine the effect of VR-based postural control training on the gait ability of patients with chronic stroke by comparing a VR treatment with conventional physiotherapy [53]. Subjects in both groups received conventional physical therapy, and only subjects in the VR group received additional VR training. This VR-based program was intended to allow for the control of the subject’s posture through visual feedback by watching their actual motion. In the within-group comparisons, participants in the VR group showed significant improvements in gait parameters (except for cadence) just after training and at the end of the follow-up period compared to the control group. Between groups, the VR group performed significantly better only in stride length (compared to the control group).

Jung et al. [50] compared a VR treadmill approach with simple treadmill training. The control group received treadmill training (without control over the slope of the treadmill), whereas the experimental group received VR treadmill training with an HMD. The subjects in the VR group achieved higher improvements (in both balance and balance self-efficacy) after the treatment compared to the control group. Both groups exhibited significant improvements after training in both balance and balance self-efficacy compared to baseline values.

Kim et al. [51] conducted a treadmill-based intervention by dividing the population into three groups. One group received functional electrical stimulation (FES) with VR during treadmill gait training, another group received FES during treadmill training, and another group received treadmill training. The VR-FES and FES groups showed greater improvements in muscle strength compared to the control group. Greater improvements were achieved in gait speed (assessed by TUG) in the VR-FES group compared to the control group. The BBS showed a significant increase within groups, but there was no difference between groups.

In the study of Lee [46], all patients participated in a general physical therapy program for four weeks. However, patients that belonged to the experimental group received additional VR-based postural control training in the same four-week period. The results showed a significant effect of time on TUG, BBS, stride length, cadence, velocity, and step length for both paretic and nonparetic sides. A significant group × time interaction effect was only achieved in step length, walking velocity, and stride length on both the paretic and nonparetic sides.

Crosbie et al. [47] designed virtual tasks to simulate several upper limb tasks, such as reach to target and reach and grasp [54]. The task allowed the modulation of several parameters, such as the speed of stimulus, the height of objects, and the distance of objects, according to progress in the subject’s performance. After nine treatment sessions (three weeks), no significant changes after training were obtained.

Ögün et al. [45] designed four different games focused on the gripping and handling of objects with the arm and forearm while using a device (HMD) that covered both eyes and ears. The control group received conventional therapy comprising the same tasks as those used in the control group. The control group also used the HMD but without any upper limb interaction (sham VR therapy only for masking purposes). In the inter-group comparison, the authors found significant differences in favor of the VR group in all of the outcome measures (FMUE, ARAT, FIM PASS-BADL, and PASS-IADL).

#### 3.4.1. The Timed Up and Go (TUG) Test

**Figure 2:**
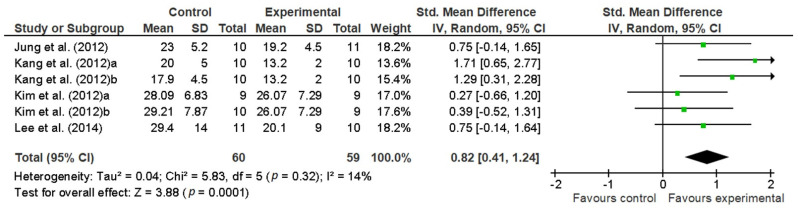
Pooled standardized mean differences and overall effect size (95% CI) on the TUG test immediately after the intervention.

Four studies were included in the meta-analysis for the effect of VR interventions on TUG scores [46,48,50,51]. Kang et al. [48] examined the effects of an immersive VR system that provided speed-modulated optic flow together with treadmill training. Significant improvements were reported in comparison to two control groups (treadmill training and conventional therapy, respectively). In the study of Kim et al. [51], the three groups performed treadmill gait training, but functional electrical stimulation (FES) was received by the immersive VR group and one of the treadmill groups. The results obtained for the TUG test showed an important overall effect size (0.82) [41] in favor of the groups receiving immersive VR therapies compared to the groups who received conventional therapies (control groups). Figure 2 depicts the pooled results. No important statistical heterogeneity was observed (I^2^ = 14%).

**Figure 3:**
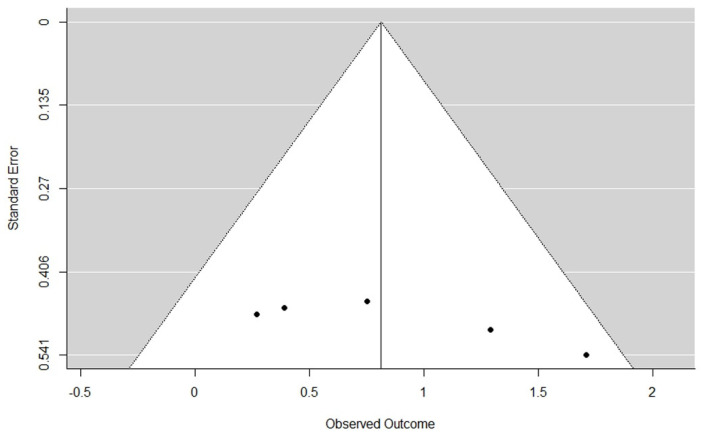
Funnel plot for the TUG measure.

Since all of the studies included in the meta-analysis were published papers, we tested whether publication bias against null results for the TUG measure could be a bias source in the effect size estimates obtained in our meta-analysis. The preliminary inspection of the funnel plot does not show asymmetry (Figure 3). The rank correlation test (Kendall’s tau = 0.2857, *p* = 0.4363) showed non-significant differences when testing the null hypothesis of homogeneity. Therefore, taking these results into account and on a reasonable basis, we can discard publication bias as a serious threat to the validity of TUG results.

#### 3.4.2. The Functional Reach Test (FRT)

**Figure 4:**
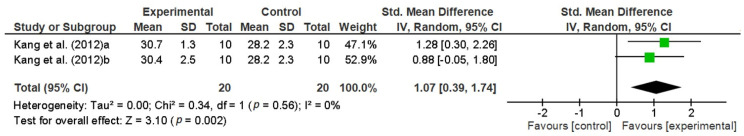
Pooled standardized mean differences and overall effect size (95% CI) on the FRT test immediately after the intervention. (a) Treadmill with optic flow versus control group. (b) Treadmill group versus control group.

The functional reach test (FRT) measures the subject’s stability by assessing the maximum distance to which a subject can reach forward while standing in a fixed position. The FRT provides a reliable and valid assessment of standing balance [55]. In the study of Kang [48], the effect size achieved by the treadmill group with optic flow compared to the control group was large (ES = 1.28). The treadmill group compared to the control group (0.88) achieved a lower effect. The results are depicted in a forest plot to observe the differences between groups (see Figure 4). However, there was no significant effect size between the treadmill group with optic flow compared to the treadmill group (ES = 0.14, CI: −0.73, 1.02).

#### 3.4.3. The Six-Minute Walk Test (6MWT)

**Figure 5:**
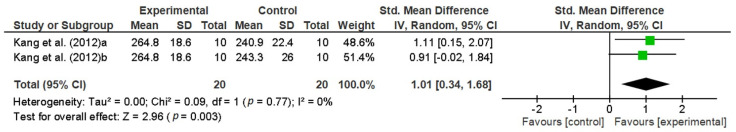
Pooled standardized mean differences and overall effect size (95% CI) on 6MWT test immediately after the intervention in Kang et al.’s study. (a) VR treadmill group versus control group. (b) VR treadmill group versus treadmill group.

The study of Kang et al. [48] found that the distance of the six-minute walk test (6MWT) in the treadmill with optic flow group showed significant improvement compared to the treadmill and control groups, respectively. The achieved effect size between the VR treadmill group and the control group was large (ES = 1.1), and the effect size between the VR treadmill group and the treadmill group was also large (ES = 0.91) [41]. Figure 5 depicts the pooled results. Jaffe et al. [52] also measured the 6WMT and reported an improvement greater than 5% in both groups (VR group and control). Nevertheless, subjects belonging to the VR group showed a greater percentage of retention in the 2-week follow-up period compared with the post-training.

#### 3.4.4. The 10-Meter Walk Test (10MWT)

**Figure 6:**
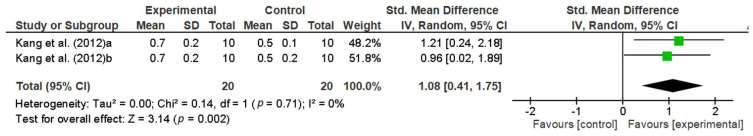
Pooled standardized mean differences and overall effect size (95% CI) on 10MWT immediately after the intervention in Kang et al.’s study. (a) VR treadmill with optic flow versus control group. (b) VR treadmill with optic flow group versus treadmill group.

This test is used to assess walking speed over a short distance (in meters per second). The study of Kang et al. [48] reported that the treadmill with optic flow group showed significant improvements compared to the treadmill and control groups post-treatment (see Figure 6). In addition, Jaffe et al. [52] measured gait speed over 10 m but only reported percentage improvements. The group receiving the VR intervention training achieved significantly faster walking speed compared to the control group.

#### 3.4.5. The Action Research Arm Test (ARAT)

**Figure 7:**
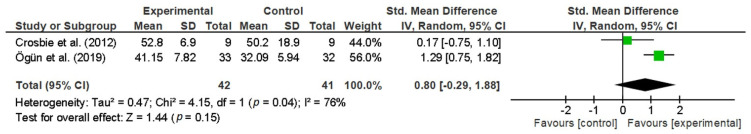
Pooled standardized mean differences and overall effect size (95% CI) on the ARAT immediately after the intervention.

Crosbie et al. [47] used the ARAT and upper limb MI to determine upper limb function. Both groups (control and VR) achieved small (and non-significant) changes in their arm impairment and activity levels. The authors stated that the measures were not sensitive enough to detect either small or moderate changes in the participants and suggested that a larger trial be performed in order to achieve reasonable significant improvements in both MI and ARAT for a significance level of *p* = 0.05 and a statistical power of 85%. On the other hand, Ögün et al. [45] found that both groups (VR and control) achieved significant improvements in upper extremity function and functional independence, respectively. Nevertheless, the group-wise comparison showed that the VR group had greater improvements (ARAT and FMUE tests) compared to the control group. According to the authors, FMUE and ARAT scores improved in a way that was considered clinically important. The authors used a sample of 65 subjects, 32 belonging to the control group and 33 belonging to the experimental group. Figure 7 shows the pooled standardized mean differences between groups and the overall effect size on the ARAT immediately after the intervention. It can be observed that there is great heterogeneity (I^2^ = 76%) between studies due to the large sample used in the study of Ögün et al. [45] compared to that in the study of Crosbie et al. [47]. Nevertheless, it is worthwhile to mention that a large ES was achieved by the subjects in the former study (ES = 1.29).

#### 3.4.6. The Fugl-Meyer Upper Extremity (FMUE)

**Figure 8:**
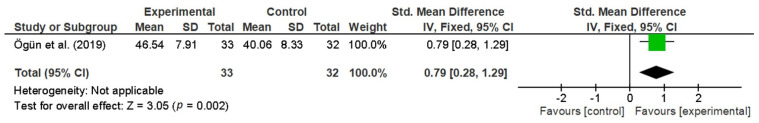
Pooled standardized mean differences and overall effect size (95% CI) on the FMUE test immediately after the intervention.

The only study assessing the Fugl-Meyer upper extremity (FMUE) was the study of Ögün et al. [45]. They found a significant increase in the FMUE scores after the intervention for the experimental group compared to the control, exhibiting a large effect size (0.79) according to Cohen [41]. Figure 8 depicts the standardized mean difference and overall effect size on this parameter.

#### 3.4.7. Other Measures

**Figure 9:**
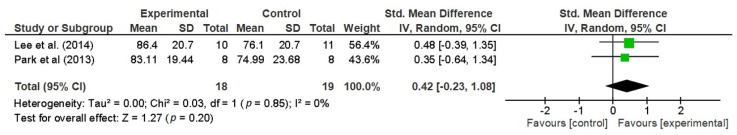
Pooled standardized mean differences and overall effect size (95% CI) on the stride length (paretic side) immediately after the intervention.

The activities-specific balance confidence (ABC) scale was only evaluated by one study [50], in which a small effect size (not statistically significant) was obtained in favor of the experimental group. We found some other measures of gait parameters. Park et al. [53] found no significant improvements in cadence in the VR group compared to the control group. Lee et al. [46] also found no significant improvements in the time × group interaction effects on cadence, despite having a significant improvement in the VR group at the end of the intervention. The study of Jaffe et al. [52] showed a slight change in cadence (not significant). Velocity was measured in two studies [46,53], showing no significant improvements and a non-significant overall effect. Jaffe et al. [52] found that the group receiving the VR training achieved a longer (and significant) stride length on the paretic side for the fast-pace walking evaluation tests. Lee et al. [46] and Park et al. [53] also measured this parameter, but the overall effect was not significant (Figure 9).

## 4. Discussion

The main goal of this systematic review and meta-analysis was to evaluate the evidence regarding the use of fully immersive VR interventions to improve motor functions on adult stroke populations. To the authors’ best knowledge, this is the first systematic review to separately analyze the effects of immersive VR rehabilitation systems on stroke populations. Results from the meta-analysis demonstrate that patients who received immersive VR treatments largely improved compared to those receiving traditional or conventional therapies. The effect sizes in terms of standard mean differences were large [41] on most scales for both lower and upper extremities.

The results obtained for the TUG test showed an important overall effect size (0.82) [41], with an absolute average value of nearly 5 scale points. This means that patients who received immersive VR therapies had more important (and statistically significant) improvements compared to the patients who received conventional therapies (control groups). Our findings are comparable to and even greater than the results obtained in previous and recent meta-analyses in which the majority of VR interventions were non-immersive. In their meta-analysis, Liz et al. found moderate improvements in subjects receiving the VR intervention (absolute mean difference of −1.62, 95% confidence interval of −3.07 to −0.16, *p* < 0.05, I^2^ = 24%). The degree of homogeneity was comparable to that found in our study [32]. Corbetta et al. [33] also found statistically significant benefits for mobility in the TUG test (absolute mean value of 2.3 s) when VR training replaced traditional rehabilitation (in part or completely).

Iruthayarajah et al. [34] found a mean difference in the overall effect size of 2.49 in their meta-analysis. The overall effect size obtained in our meta-analysis is an absolute value of around 5, which doubles the previous result. Our results parallel these findings and add to the existing literature evidence of strong positive effects of using immersive VR interventions. However, this greater overall effect size showed by our meta-analysis may have been overestimated due to the smaller subject sample used.

### 4.1. VR Treadmill and Feedback

In general, interventions with treadmills have shown positive effects on gait and balance [48,50,51,52]. The obtained results for 6MWT, 10MWT, and TUG scores confirm this. Treadmill training has previously been reported to be an effective method for restoring gait function by providing a task-oriented repetitive practice [34,56]. This task-oriented and repetitive activity is believed to activate the cerebral cortex and, subsequently, to evoke motor learning [50]. In addition, an improved balancing ability and effective motor learning with a faster gait speed occur [57]. In particular, VR treadmill training has better effects on balance skills compared to traditional treadmill training. Yang et al. compared traditional treadmill and VR treadmill training, and they found that the VR treadmill practice improved balance skills in the medial-lateral direction and during sit-to-stand transfers better than traditional training did [58].

Nevertheless, VR treadmill training usually involves the addition of cueing and feedback in different modalities (visual, audio, and vibrotactile). Therefore, in addition to the effects of the treadmill, the introduction of feedback and cueing to treatments has been beneficial for the outcomes of studies. VR allows users to interact with a multisensory simulated environment and receive “real-time” feedback on performance [35]. All of this sensory information allows the central nervous system to better control the position and orientation of body parts, promoting the adaptation to the external environment [59].

Practice in VR environments has shown the importance of the perception of self-motion in controlling different locomotion parameters, such as posture or gait. The real-time nature of all feedback and cueing modalities plays an important role in immersive VR interventions. In the study of Jaffe [52], the visual (the presentation of the foot as it approached the object), vibrotactile (error detection), and auditory feedback in the virtual environment provided several ways to warn the subject of a collision. For example, lateral views of the legs gave subjects visual cues, leading to a better performance. In the study of Kang [48], the modulation of optic flow (OF) provided an effective training method for balance and gait rehabilitation. Optic flow is the pattern of the visual information (direction and speed) generated by the relative motion between a patient’s eye and the surrounding environment [60], and when the patient identifies the incongruity between optic flow and their proprioceptive information from the lower extremities, walking speed is adjusted to diminish the incompatibility [61,62]. Therefore, the manipulation of optical flow characteristics can cause changes in locomotion patterns. These good results are in line with those obtained in previous studies that were conducted to investigate the effects of the velocity of optic flow on both postural control and gait variability and walking speed.

Pickhinke et al. investigated the effects that the variation of optical flow speed may have on postural control during locomotion. They suggested that postural control may be affected when the perception of self-motion becomes unpredictable [63]. Some other studies have previously established that the modulation of optic flow can change locomotor performance by reducing gait variability [64,65,66,67]. Lamontagne et al. [68] compared the modulation of walking speed in response to OF speed changes between persons with stroke and healthy controls and concluded that VE manipulation of the OF could be used to foster volitional changes in walking speed. Stroke patients were able to increase walking speed when presented with slower OFs. This is in line with previous studies that indicated that decreasing the rate of optic flow relative to normal walking speed is correlated with an increase in walking speed in a healthy individual [69]. Therefore, the manipulation of optic flow speed using virtual reality technology could be implemented in a gait rehabilitation intervention to promote faster walking speeds after stroke.

Previous studies have examined the effect of visual feedback on brain reorganizations in stroke [70] and the role it may play in the recovery of locomotion in patients with chronic stroke [71]. Furthermore, the literature includes many studies that report that visual feedback motivates patients to increase their concentration [72,73] and improves the balance of stroke patients [74,75]. This adds to the motivation and psychological stability that the use of VR provides by itself to stroke patients [13].

As far as immersive VR interventions for upper extremity rehabilitation are concerned, the study of Crosbie [47] is in line with the findings of the studies by Crosbie et al. [76] and Henderson et al. [7] since it reports very limited evidence for virtual reality-based therapies. One possible explanation for the poor results obtained by Crosbie et al. [47] involves the fact that the requirement to make objects appear reachable is a big challenge in the creation of VEs [77]. Virtual spaces characteristically appear to the viewer as smaller than physical spaces [78]. This decreasing phenomenon has been previously reported [78,79]. Liebermann et al. reported such limited depth-perception in the 2D projection of the environment in their study [80]. Piggott et al. reported some of the problems that occur when using 2D VR systems [81], demonstrating that all subjects had a tendency to decrease their wrist extension (increasing the elbow extension at the same time). One plausible reason was related to the missing depth cues in a 2D environment. They also suggested that fully immersive VR environments need to take into account depth cues and the type of haptic feedback, provide haptic cues for object collision, and emulate the performance of tasks in natural environments. Despite the good results obtained in the study of Ögun et al. [45], the authors also pointed to the provision of multisensory feedback (auditory, visual, and tactile) in virtual reality tasks as a way to enhance cortical reorganization and, therefore, result in an improvement in motor function.

With regard to previously reported adverse incidences related to the use of immersive VR equipment (visual disturbances, nausea, and headache) [2,82], only one study reported some discomforts [47]. Two people experienced side-effects (transient dizziness and headache) in the study of Crosbie et al. [47]. Jaffe did not report any HMD adverse effects in their study, in spite of the fact that one subject had previously experienced episodes of claustrophobia at home [52]. The dropouts reported in the study of Ögün et al. [45] were the result of compliance issues. In spite of the fact that visual disturbances, nausea, headache, or other discomforts in VR-treated subjects seem to have been overcome [83], we still find some examples where they are present. Tsoupikova et al. [84] reported several occurrences, such as mild eyestrain and transient nausea that arose during pilot studies with an HMD. These occurrences, along with complaints about comfort, prompted them to switch from the HMD to two large displays. They found that environmental cues could be at least as effective as stereoscopic vision in providing perceived size constancy of a virtual object as it is moved to different depths in a VR environment. Consequently, a rich sense of depth was provided even without use of the HMD.

### 4.2. Limitations

This review has limitations that are worth mentioning. The final number of studies meeting the inclusion criteria was modest. As far as the outcome measures were concerned, there was no uniformity among the studies; as a result, some gait parameters were not meta-analyzed. Among the analyzed studies, we found great variability in both the duration and intensity of the treatment. Larger and more intense treatments do not always lead to greater improvements, as in the study of Lee et al. [46]. Furthermore, the sample size was small in all of the included studies (10 subjects per group on average), which may lead to reduced statistical power.

Despite the collection of RCTs to reduce the bias risk, we think it is necessary to include a larger number of participants, together with a wider range of population groups. In our review, most of the studies were carried out in one single country, which suggests that multicenter studies located in different geographic locations are needed in order to obtain results with a greater degree of generalizability to any setting or population. In the same way, the results of the FRT, 6MWT, and 10MWT (Figure 4, Figure 5 and Figure 6, respectively) are all based on publications from one group.

A common feature stated in many reviews is the fact that the power and design of studies are limited, so the support that they offer remains weak to moderate in quality. The long-term effects of immersive VR interventions have not been evaluated in most of the included studies. The results of this meta-analysis cannot be generalized to the acute or sub-acute stage of hemiparetic stroke, since all of the interventions were intended for chronic stroke patients.

### 4.3. Implications for Research

Our meta-analysis suggests that there are several important implications for further research. Firstly, the primary studies show some deficiencies as far as the reporting of gait and motor function parameters is concerned. Several studies present the information as a percentage ratio of pre- and post-intervention in some of the outcomes, which makes it difficult to interpret the results and compare them to the effect sizes of the rest of the studies. We strongly recommend reporting the effect size in terms of standardized mean differences, since it is a more reliable measure of the real effect of the interventions.

Secondly, the short periods of time used in most of the interventions suggest the need for trials with larger treatments periods for a better evaluation of the functional gains. It is important to bear in mind the fact that differences in motor severity and chronicity should be related to dose requirements. For that reason, further research should be oriented to that population in acute or sub-acute stages.

Another aspect that should be improved in the design of these studies is the obtaining of follow-up data for the treated and control groups. The long-term effects of VR have not been explored yet, in contrast to other forms of stroke rehabilitation. In fact, only a small number of the studies included in our meta-analysis had established follow-up analyses [47,52,53], and the periods were very short. This limitation of the primary studies, limits, in turn, the possibilities of using a meta-analysis to investigate how the achieved benefits of immersive VR therapies evolve over time. For this purpose, it would be suitable to obtain comparisons between the control group and the experimental group over long enough periods of time to evaluate the benefits of immersive VR treatments. Therefore, this necessary quantification of the residual benefits in the short and medium term requires further studies. This is of particular relevance in chronic stages of the disease. For the abovementioned reasons, further studies are also needed to elucidate if better performance is due to larger treatments rather than the use of immersive VR systems.

Regarding the lack of depth-perception in upper limb interventions, there is so much work to be done, since there is a considerable lack of fully immersive VR interventions. Subramanian and Levin compared the effects of viewing a virtual environment via HMD and large screen projection systems (SPS) on upper limb pointing movements. They found that the movements performed by the stroke subjects were less precise. Furthermore, they realized that there was an important vertical directional error when using the HMD. The authors remarked that wearing HMDs may result in neck discomfort, affecting distance estimation. Consequently, they posited that large screen projection systems (SPS) are more comfortable and effective than HMDs for rehabilitation post-stroke [85].

On the other hand, presence seems to be a very important feature when dealing with immersive VR systems as far as performance is concerned [27,28], but it has been seldom assessed in the literature. Therefore, the sense of presence of participants involved in immersive VR interventions should be addressed to elucidate the effect of wearing an HMD in rehabilitation processes [85].

In a recent study, Borrego et al. [86] assessed presence by means of two questionnaires, the original Slater-Usoh-Steed Questionnaire [87] and a modified version of the Presence Questionnaire [88]. The authors evaluated an HMD-based VR system (enabling head tracking) that was shown to elicit a significantly higher sense of presence than Cave Automatic Virtual Environment (CAVE) systems. This promotion of a higher sense of presence plays an important role in enhancing the ecological validity of immersive VR rehabilitation applications. Future research in this direction could give us some more information about the sense of presence and its impact on participant compliance with exercise and final performance. This would justify the use of HMDs in VR interventions.

Another important issue that has been pointed out by different authors is related to the transfer of positive results achieved by subjects to activities of daily living (ADL) [89]. Although some progress has been made in the demonstration of the transfer of abilities and skills acquired within VE to real-world performance [5,90,91], several studies (non-immersive) intended for the stroke population have reported an inherent difficulty in achieving such a transfer [92,93]. Therefore, it is very important to ascertain if the induced improvements are ecologically valid at home or in community environments [94].

Finally, due to the difficulty of incorporating unpublished studies into a meta-analysis in this field, it is highly recommended to routinely examine whether publication bias may be a threat to the validity of results.

### 4.4. Implications for Practice

The findings from our review suggest that the use of immersive VR in motor virtual rehabilitation is a promising intervention when used in stroke populations. VR has the ability to create a rich virtual environment by modulating both the intensity of the task and sensory feedback to provide the most appropriate and individualized motor training [15,95]. Virtual cueing and feedback are important in the sense that they normalize the internal cueing deficit to prepare the patient for the next movement while compensating for any malfunctioning sensory integration [96].

The richness of stimuli provided by immersive VR (cueing and feedback) has been successfully exploited by some of the interventions [48,51,52,53], which has led to very large effect sizes in the TUG, FRM, and 10MWT tests. The TUG overall effect size is greater than the results obtained in previous and recent meta-analyses in which the majority of VR interventions were non-immersive.

On the other hand, in spite of the fact that the study of Lee et al. [46] used visual feedback, the addition of VR-based training did not result in a better performance on the TUG test compared to controls (who received conventional therapy only). A relatively short treatment duration as well as the small sample size could lead to this lack of significant treatment effect on the TUG test. This is in line with previous studies, like the one developed by Walker et al. [14], who found no significant between-group differences when visual feedback was added in the experimental group in a balance training intervention of acute stroke patients. It is worth taking into account the preferred modality of the cue or feedback for every patient in order to improve the rehabilitation process [97], as well as the point at which it should be applied in the rehabilitation program. For these reasons, greater improvements might be achieved by further customizing the applied cueing and feedback for every single patient [98]. Clinicians should take into account that the appropriateness of the use of these stimuli depends on the person’s rehabilitation goals and preferences, in the interest of improving the patient performance [89].

Although there are several studies that have claimed that the HMD is effectiveness in inducing vestibulo-ocular reflex (VOR) gain adaptation in subjects with chronic dizziness [99] or in providing optokinetic stimulation and visual–vestibular interaction for subjects with balance disorders [100], there some other semi-immersive interventions that have questioned the use of HMDs. Kim et al. [94] used an IREX VR system to enhance the motivation and static and dynamic balance performance associated with gait in patients with stroke. Within this system, subjects were free to move in the real world while manipulating virtual objects in the 3D virtual environment without requiring a heavy HMD or other peripheral devices. This greater freedom of mobility, as well as the non-necessity of heavy and expensive devices, was remarked upon in [70,94]. Fischer et al. found that it was difficult for some subjects to lift their arms while looking at the virtual environment due to the limited field of view (FOV) of the HMD used. The authors remarked that an HMD with a wider field of view could reduce these demands on motor control [101]. These limitations on FOV, together with the weight of the HMD, are factors that can lead to an incorrect distance estimate, and it could be a confounding factor that impacts distance perception when upper limb movements are performed [102].

Finally, in terms of practical considerations, we are not able to state whether the cost of the immersive technology justifies the benefits. We have seen that the use of immersive VR interventions provides improvements in some gait parameters, but we cannot state that immersive systems perform better than non-immersive or semi-immersive systems. Therefore, there is still work to be done to determine whether the benefits we are targeting are clinically worthwhile.

## 5. Conclusions

This review and meta-analysis has shown some improvements in balance and gait in post-stroke patients when immersive VR interventions are applied and compared to conventional rehabilitation therapies. The results confirm the effects of interventions and justify further clinical trials (positive effects but with limitations). Although the number of RCTs using immersive VR systems for stroke rehabilitation is growing, there are still unresolved questions to address to determine whether the benefits are clinically worthwhile. This fact should encourage future immersive interventions with larger and multicenter populations to elucidate whether this kind of immersive therapy could perform better than non-immersive therapies. Despite its limitations, this review encourages the use of immersive VR interventions in conjunction with traditional therapies for the recovery of motor impairments in stroke subjects. Future studies are needed with a larger number of subjects to improve the internal and external validities.

## Figures and Tables

**Table 1:** Characteristics of the included studies.

Author(s)/Year of Publication/ PEDro Score	Participants	Stroke Onset (Months)	Study Design	Control/Experimental Group Interventions	Outcome Measures	Conclusions
	Age (yrs ± SD)					
	Gender (M, F)					
Jaffe et al. (2004) [52]/ PEDro: 4/10	*N* = 20	Control: 42.9 ± 30.1	RCT	Control: stepping over real foam objects in a hallway (60 min, 3/week × 2 weeks)	Gait endurance: 6MWT	The VR training achieved higher improvements in velocity compared to control training
	OG group (*N* = 10)					Subjects showed clinically meaningful changes in stride length, velocity, obstacle clearance capacity, and walking endurance
	Age: 63.6 ± 8.3					
	Gender: (7 M, 3 F)					
	VR group (*N* = 10)					
	Age: 58.2 ± 11.2				Gait kinematics: spatiotemporal gait parameters (walking velocity, cadence, and stride length)	
	Gender: (5 M, 5 F)	Exp: 47 ± 27.5		Experimental: stepping over virtual objects on a treadmill (60 min, 3/week × 2 weeks)	Obstacle clearance test, balance test	
Jung et al. (2012) [50]/ PEDro: 5/10	*N* = 21	Control: 15.4 ± 4.7	RCT	Control: treadmill walking training (30 min, 5/week × 3 weeks)	Balance (TUG)	There were significantly higher improvements in TUG and ABC in the VR treadmill-training group compared to the control group.
	Control group (*N* = 10)					Significant increases in TUG and ABC in both groups after training.
	Age: 63.6 ± 5.1					Improvements seen in the experimental group were significantly larger than the control group.
	Gender: (6 M, 4F)					
	Exp. group (*N* = 11)					
	Age: 60.5 ± 8.6					
	Gender: (7 M, 4 F)	Exp: 12.6 ± 3.3		Experimental: treadmill walking in a virtual outdoor environment (30 min, 5/week × 3 weeks)	Balance self-efficacy (ABC scale)	
Park et al. (2013) [53]/ PEDro: 5/10	*N* = 16	Control: 135 ± 54.4	RCT with follow-up	Control: two administrations of conventional rehabilitation (60 min, 5/week × 4 weeks) + (30 PT min, 3/week × 4 weeks)	Functional gait ability (10MWT)	Subjects in the VR group showed a significant improvement (except for cadence) after training and at the follow-up (compared to the control group).
	Control group (*N* = 8)					Within groups, the VR group demonstrated greater improvements in stride length (only) compared to the control group
	Age: 48.75± 8.81					No significant differences found in other parameters
	Gender: (5 M, 3 F)					
	Exp. group (*N* = 8)					
	Age: 46.25 ± 6.84					
	Gender: (6 M, 2 F)	Exp: 139.5 ± 53.3		Experimental: conventional rehabilitation (60 min, 5/week × 4 weeks) + VR-based postural control exercises (30 min, 3/week × 4 weeks)	Spatiotemporal gait ability (velocity, cadence, step length, and stride length)	
Kang et al. (2012) [48]/ PEDro: 7/10	*N* = 30	TOF group: 14.1 ± 4.4	RCT	Control: Conventional rehabilitation (30 min × 5/week × 4 weeks) + stretching added ROM exercises (30 min, 3/week × 4 weeks)	Balance (TUG, FRT)	Treadmill using optic flow speed modulation improved the balance and gait significantly compared to the control group
	TOF group (*N* = 10)	Treadmill group: 13.5 ± 4.0			Gait (6MWT, 10MWT)	
	Age: 55.9 ± 6.5					
	Gender: (6 M, 4 F)	Control group: 15.1 ± 7.4				
	Treadmill group (*N* = 10)					
	Age: 56.3 ± 7.6					
	Gender: (4 M, 6 F)					
	Control group (*N* = 10)					
	Age: 56.1 ± 7.8			Treadmill group = Conventional rehabilitation (30 min × 5/week × 4 weeks) + treadmill training (30 min, 3/week × 4 weeks)		
	Gender: 6 M, 4 F			TOF group (Exp.): Conventional rehabilitation (30 min × 5/week × 4 week) + treadmill walking with optic flow (30 min, 3/week × 4 weeks)		
Crosbie et al. (2012) [47]/ PEDro: 8/10	*N* = 18	Control: 11.7 ± 7.8	RCT with follow-up	Control: conventional therapy (30–45 min, 3/week × 3 weeks)	ARAT, upper limb motricity index	Neither small nor moderate changes were detected by measures
	Control group (*N* = 9)					No significant improvement in either the control or intervention groups
	Age: 66.4 ± 7.4					Transient dizziness and headache experienced by two participants in the VR group
	Gender: (5 M, 4 F)					
	VR group (*N* = 9) Age: 56.1 ± 14.5					
	Gender: (5 M, 4 F)	VR group: 10 ± 6.4		Experimental: specific upper limb VR tasks (reach to target, reach and grasp) (30–45 min, 3/week × 3 weeks)		
Lee et al. (2014) [46]/ PEDro: 7/10	*N* = 21	Exp: 11.7 ± 4.5	RCT	Control: general physical therapy focused on postural control training (30 min, 5/week, 4 weeks)	BBS, TUG, gait velocity, stride length, cadence, and step length	The addition of VR-based training conveyed to significant improvement in the following gait variables (step length, stride length, and gait velocity) compared to general physical therapy treatment only (control group)
	Control Group (*N* = 11)					
	Age: 54.0 ± 11.9 Gender: (6 M, 5 F)					No significant time × group effect in both TUG and BBS.
	AR group (*N* = 10) Age: 47.9 ± 12					
	Gender: (8 M, 2 F)	Control: 11 ± 4.7		Experimental: general physical therapy (30 min, 5/week × 4 week) + VR-based postural control training (30 min, 3/week × 4 weeks)		
Kim et al. (2012 [51])/ PEDro: 4/10	*N* = 28	Control: 10.4 ± 3.1	RCT	Control: general physical therapy (30 min, 5/week, 8 weeks) + treadmill gait training (20 min, 3/week, 8 weeks).	TUG, BBS, muscle strength	VR-FES and FES groups showed greater improvements in muscle strength than control group
	Control group (*N* = 9)			FES group: general physical therapy (30 min, 5/week, 8 weeks) + treadmill gait training + FES (20 min, 3/week, 8 weeks)		Greater improvements in gait speed (assessed by TUG) in VR-FES group than control group
	Age: 49.11 ± 11 Gender: (6 M, 3 F)			VR + FES group: general physical therapy (30 min, 5/week, 8 weeks) + treadmill gait training + VR + FES (20 min, 3/week, 8 weeks)		Significant improvements in BBS in all groups
	FES group (*N* = 10) Age: 51.5 ± 12.9 Gender: (5 M, 5 F)					
	VR-FES group (*N* = 9)					
	Age: 47.4 ±8.4	FES group: 9.2 ± 2.7				
	Gender: (6 M, 3 F)	VR-FES group: 9.7 ± 4.2				
Ögün et al. (2019) [45]/ PEDro: 6/10 *	*N* = 65	Control: 15.37 ± 9.77	RCT	Control: conventional upper extremity active exercises focused on gripping and handling (45 min, 3/week, 6 weeks) + passive VR therapy (15 min, 3/week, 6 weeks)	FMUE, ARAT, FIM, PASS-IADL, PASS-BADL	Significant improvements in FMUE, ARAT, FIM, and PASS scores (compared to baseline)
	Control group (*N* = 32)					
	Age: 59.75 ± 8.07 Gender: (23 M, 9 F)					
	VR group (*N* = 33) Age: 61.48 ± 10.92 Gender: (28 M, 5 F)	VR group: 14.72 ± 7.38		Experimental: task-oriented games focused on gripping and handling (60 min, 3/week, 6 weeks)		

ABC: activities-specific balance confidence scale; ARAT: action research arm test; BBS: Berg balance scale; FES: functional electrical stimulation; FIM: functional independence measure; FRT, functional reach test; FMUE: Fugl-Meyer upper extremity scale; HMD: head-mounted display; OG: real obstacle training; RCT: randomized controlled trial; ROM: range of motion; PASS-BADL: Performance Assessment of Self-Care Skills—basic activities of daily living; PASS-IADL: Performance Assessment of Self-Care Skills—instrumental activities of daily living; PE: physical environment; PT: physical therapy; 6MWT: six-minute walk test; 10MWT: 10-meter walk test; TOF: treadmill with optic flow; TUG: timed up and go test; VE: virtual environment; VR: virtual reality; SPS: screen projector system. VR + FES: virtual reality + functional electrical stimulation. (*) The score was obtained by consensus between authors because it is not yet in the PEDro database.

## Data Availability

Not applicable.
